# Orientation and emigration of larval and juvenile amphibians: selected topics and hypotheses

**DOI:** 10.1163/15685381-bja10081

**Published:** 2022-02-17

**Authors:** Lukas Landler

**Affiliations:** University of Natural Resources and Life Sciences (BOKU), Institute of Zoology, Gregor-Mendel-Straße 33, 1180 Vienna, Austria

**Keywords:** amphibian conservation, metamorphosis, natal dispersal, navigation, philopatry, y-axis

## Abstract

Most amphibians have a complex life cycle with an aquatic larval and an adult (semi-) terrestrial stage. However, studies concerning spatial behaviour and orientation mainly focus on either the aquatic larvae or the adult animals on land. Consequently, behavioural changes that happen during metamorphosis and the consequences for emigration and population distribution are less understood. This paper aims to summarize the knowledge concerning specific topics of early amphibian life history stages and proposes several testable hypotheses within the following fields of research: larval and juvenile orientation, influences of environmental and genetic factors on juvenile emigration, their habitat choice later in life as well as population biology. I argue that studying larval and juvenile amphibian spatial behaviour is an understudied field of research, however, could considerably improve our understanding of amphibian ecology.

## Introduction

Amphibians are among the most threatened taxa in the world, about 40% of the species in this group are at risk of extinction (Stuart et al., 2004; Vié, Hilton-Taylor, and Stuart, 2009; Bishop et al., 2012). Habitat destruction and loss of connectivity as well as deadly pathogens are leading to rapid decline of amphibian diversity and local population sizes (Becker et al., 2007; Ficetola et al., 2015; Lips, 2016). The current paper focuses on amphibians with a specific complex life cycle, occupying at least two habitat types in their life history: an aquatic breeding habitat for spawning and (free swimming) larval development, and a terrestrial habitat for foraging (Wilbur, 1980). However, several amphibian taxa, especially in the neotropics, do not follow this pattern and are therefore not considered in here. Some deviations of such pattern (i.e., no free swimming larvae) can be related to different levels of parental care taking which are widespread and evolved many times in the amphibian evolutionary history (Furness and Capellini, 2019; Schulte et al., 2020). Such care taking can range from tadpole attendance/transport (e.g., poison frogs (Weygoldt, 1980; Pašukonis et al., 2017) to brooding (e.g., Darwin’s frog; Cabeza-Alfaro et al., 2021) and vivipary (e.g., Alpine salamander; Guex and Chen, 1986). Other amphibian species are fully aquatic and reach maturity in their aquatic larval form; one of the best studied examples is the Mexican axolotl (Tompkins, 1978; De Groef, Grommen, and Darras, 2018), although such life history strategy is wide-spread in urodeles (Johnson and Voss, 2013). However, most amphibians in temperate regions can be categorized as having a complex life cycle with an aquatic (free swimming) larval development and a (semi-) terrestrial adult stage.

Inherent to such life cycles are breeding migrations, which in many cases require amphibians to navigate surprisingly long distances. Migration distances up to 15 km have been reported (Tunner and Karpati, 1997). Adult migration and navigation of amphibians was the subject of numerous reviews (Sinsch, 1990, 1991, 2006) and here I only want to summarize several essential points: Environmental conditions influence the timing of migration, however, only within defined seasonal limits; amphibians can use olfactory, acoustic, visual and magnetic orientation cues; for several species it has been shown that they migrate to the same areas year after year (‘site fidelity’).

There are several obvious reasons why spatial behaviour of adult amphibians is much better studied than that of juveniles: adults are usually larger and therefore it is possible to use tracking devices such as radiotelemetry; their survival rate is higher, this means capture-recapture studies are more suitable; while adult migrations are often tightly timed and happen over defined periods, juvenile movements may appear more stochastic and timing of such are not well understood (Rothermel, 2004). Nevertheless, understanding the spatial behaviour of juvenile amphibians may be critical for predicting species distribution, extinction threats and informing conservation efforts (e.g., Petrovan and Schmidt, 2019). For instance, the habitats juveniles experience, likely influence the animals’ decision to disperse or to stay (e.g., Denoël et al., 2018; Cayuela et al., 2020). The aquatic larvae as well as the dispersing juveniles have much higher mortality rates than the adult stage (Lutz, 1948; Rothermel, 2004). It is this stage where amphibians are most susceptible to pathogens, such as the chytrid fungus (Langhammer et al., 2014). While adult spring migration is, at least occasionally, protected by road mitigation efforts, such as drift fences and temporary conservation measures, juveniles leaving the breeding habitats are often left unprotected (Petrovan and Schmidt, 2019).

From a conservation standpoint, understanding juvenile emigration is at least as important, as the reoccurring migration of adults, many life history decisions related to space use (e.g., where to settle) happen at this stage (Petrovan and Schmidt, 2019). In this paper I am synthesizing the knowledge of larval and juvenile spatial behaviour ecology, mainly focused on sensory aspects. Based on existing evidence, I formulate several testable hypotheses, which would substantially improve our understanding of amphibian early life history. It is important to add that this is not a review of the amphibian dispersal literature, several excellent reviews on this topic have been published over the recent past (see above and Smith and Green, 2005; Baguette et al., 2013; Sinsch, 2014; Joly, 2019; Cayuela et al., 2020). This paper focusses on a few specific subjects (i.e., larval/juvenile orientation, influence of natal habitat and landscape), which are often overlooked in the context of amphibian ecology and life history. My goal is to connect the existing evidence on these issues with hypotheses and research questions regarding amphibian spatial and population ecology.

## Larval and juvenile orientation in their natal habitat

During the larval stages, most amphibians suffer from high predation risk (Lutz, 1948), which leads to strong adaptive responses of amphibians in the presence of predators. In numerous studies it has been shown that predators in the aquatic habitat (such as dragon fly larvae or fish) will dramatically alter the morphology of larvae as well as the general behaviour (e.g., microhabitat selection and activity) (e.g., Benard, 2004; Orizaola et al., 2013). For instance, larvae can reduce swimming activity or choose predator-free parts (i.e., deeper water) of their habitat to avoid predation. Typically, along a water depth gradient, the shallow/sun exposed areas provide nutrients, more dissolved oxygen and warmer water (Nie, Crim, and Ultsch, 1999) the deeper and/or covered areas could provide refuge from predators (see discussion in Tomson and Ferguson, 1972; Diego-Rasilla, Luengo, and Phillips, 2013). Therefore, in many cases larval (but also juvenile and adult) amphibians show a directed orientation along a shallow-deep axis.

In order to describe this general phenomenon Ferguson and Landreth (1966) defined the ‘y-axis’: “If the shoreline represents the x-axis, then the y-axis extends offshore and inland at right angles to it.” It should be noted that the investigation of orientation perpendicular to shore was first investigated in marine animals following the moving shoreline, however, without definition of a term (e.g., Williamson, 1951). For amphibians, movement along the y-axis means that they will most efficiently transition between deeper and shallower water. For instance, in case a terrestrial predator approaches a pond, amphibian larvae orienting along the y-axis, can quickly hide in the deeper water after feeding in the shallow areas. In another scenario, fish could be avoided by hiding at the very shallow parts of a pond. While the functional importance seems obvious and personal observations would agree with this idea it is important to point out that, to the best of my knowledge, this has never been formally tested in controlled experiments (i.e., that y-axis orientation contributes to predator avoidance).

Y-axis orientation behaviour is easily trained and elicited and therefore has been used in many sensory studies, that were concerned with cues that amphibians use for compass orientation. In such orientation assay, animals are held for several days in tanks with an inclined bottom, leading to a deep and shallow side of the holding tank (e.g., Diego-Rasilla and Phillips, 2007). Typically, the deep end of the training tank is covered, and the animals are fed from the shallow end, this is expected to reinforce the axial training. Alternatively, animals can be taken from their habitats in the field, where they learned the direction of their natural environment (i.e., home pond) y-axis (Ferguson and Landreth, 1966; Diego-Rasilla and Luengo, 2020). Animals are then tested for their orientation in circular arenas, where animals are expected to orient along the y-axis of the training- (or natural-) shoreline, irrespectively of the testing location (Adler, 1970).

Amphibian larvae and juveniles tested in such assays, have been shown to use a variety of orientation cues. The earlier work focused on the well-developed sun compass response of amphibians (Ferguson and Landreth, 1966; Ferguson et al., 1968; Taylor, 1972). Further work indicated that such celestial cues are not perceived by the eyes, but the pineal complex, extra-ocular photoreceptors located dorsally directly under the skull (Justis and Taylor, 1976). Through this structure amphibians might be able to perceive the polarization plane of the sun and use this for their orientation response (Auburn and Taylor, 1979). Later work, which focused on the magnetic sense, showed that larval anurans and newts can be trained to a magnetic field direction using a y-axis assay (Freake et al., 2002; Rodríguez-García and Diego-Rasilla, 2006; Diego-Rasilla and Phillips, 2007). Such response is based on a light-dependent magnetic compass, which might indicate a so-called radical pair based mechanism (Freake and Phillips, 2005; Diego-Rasilla, Luengo, and Phillips, 2010, 2013, 2015).

Interestingly, the behavioural responses of larval amphibians are often axial, this means animals are orienting on the expected axis, but appear to be unable to distinguish between the direction of the shallow and deep end (Freake et al., 2002; Rodríguez-García and Diego-Rasilla, 2006; Diego-Rasilla and Luengo, 2020). However, this could be partly explained using a mixture of slightly different larval stages. The preferred direction along the y-axis has been shown to depend on the developmental stage of the animals, it switches from a preference for deeper water to a preference for shallow areas at the end of prometamorphosis (McKeown, 1968; Tomson and Ferguson, 1972; Auburn and Taylor, 1979). When the 180° orientation switch exactly happens varies and might also depend on the individual, however it could be correlated with certain physiological changes (gill reabsorption, potentially brain development, hormonal changes) in the larval development. Goodyear and Altig (1971) suggested that the y-axis orientation is continuously re-learned during metamorphosis, adapting to the topographic surrounding at each developmental stage. This would lead to a highly adaptable y-axis direction. Ultimately, juveniles might show different y-axis preferences than larvae. While it is clear that y-axis directions can be learned, it would be valuable to explore a potential inherited component. For example, do larvae reared in a featureless environment show a genetically fixed y-axis orientation? This could be the case for amphibians at large lakes with very stable y-axes (also see discussion underneath on inherited emigration direction). In fact, in other taxa, inherited y-axis orientation has been shown, for example in marine sand hoppers, which use a sun compass to orient perpendicular to shore (Pardi and Scapini, 1983).

## Topography, timing, sex-dependency, and genetics of juvenile emigration

Once the larvae completed metamorphosis, the juveniles usually stay close to or in the aquatic habitat. At this stage their orientation behaviour is comparable to adult amphibians at the breeding habitat (e.g., perpendicular orientation to the shore) (Goodyear, 1971; Adler and Taylor, 1973; Deutschlander, Phillips, and Borland, 2000). Juveniles may move back and forth along the y-axis depending on environmental conditions and day time and do not disperse for several days or weeks (Ferguson et al., 1968; Tracy, 1971; Grubb, 1973), until they decide to leave. It has been speculated that such y-axis orientation, which depends on the immediate topography, is contributing to the dispersal direction (Ferguson and Landreth, 1966). In addition, the surrounding topography, such as forest versus open areas, has been shown to strongly contribute to the dispersal behaviour in many studies. For instance, juvenile amphibians in general prefer to move into forest landscape in contrast to open fields and thereby avoiding possible desiccation risk (Rothermel and Semlitsch, 2002; Walston and Mullin, 2008; Osbourn, 2012). In many cases amphibians start exiting the water towards the open field, however, afterwards changing the direction and move towards closed canopy areas (Rothermel, 2004; Rittenhouse and Semlitsch, 2006). In addition, juveniles move straighter through open field, reducing the time they spend in this potentially dangerous landscape (Pittman and Semlitsch, 2013). Responding to the immediate surrounding by avoiding unfavourable landscapes is clearly beneficial to the emigrating amphibian. But is there a correlation between the y-axis orientation and dispersal direction, or, in other words, does the larval orientation axis contribute to the (overland) dispersal direction? Most likely, the resultant emigration direction is a combination of the acquired y-axis direction and surrounding topography. However, this idea remains to be tested.

An important question for conservation management is the timing of emigration/dispersal, in order to organize roadkill mitigation efforts, which are most often temporary (Petrovan and Schmidt, 2019). Dispersal events of juveniles often do not happen right after metamorphosis completion; however, they might wait for emigration opportunities. Already Blair (1953) described that the emigration of Mexican toads was tightly linked to rainfall and occurred as mass emigration. In several studies a relation between rain events and emigration has been shown (Buschinger et al., 1970; Chelgren et al., 2008; Gravel, Mazerolle, and Villard, 2012). However, it has been noted that some individuals will leave directly after they exit the water, while the majority waits in the proximity and leaves after rain events (Dole, 1971). Despite the longstanding question concerning the timing of emigration, evidence is scarce and further studies are clearly needed to understand the weather dependent dynamics of juvenile emigration.

In addition to environmental factors, juveniles also have certain emigration windows each year which might depend on the time of parental breeding. There are striking interspecific differences between such emigration windows. For example, wood frogs and marbled salamanders have tightly synchronized emigration movements – over few days – in contrast, yellow-spotted salamanders and red-spotted newts disperse over the course of many weeks (Timm, McGarigal, and Gamble, 2007). In addition to potential differences in the duration that animals stay at their natal habitats, where individual data are mostly lacking, such observations may also be related to differences in life history strategies. Such striking individual differences within populations would also allow to study behavioural syndromes and different behavioural strategies regarding timing of juvenile orientation and emigration events (c.f., Denoël et al., 2018; Cayuela et al., 2020). However, evidence regarding this topic is still lacking. It has been suggested that some amphibians are initiating metamorphosis after a certain duration in the water and therefore leaving the water with variable body sizes, while others may maximize their resource allocation before metamorphosis and leave at same size but variable durations (Semlitsch and Wilbur, 1988; Paton and Crouch, 2002). Such fitness trade-offs would be a fruitful avenue of research, and likewise inform conservation management about dispersal windows for certain species. It has been shown that behavioural syndromes are, at least to a certain degree, stable during metamorphosis and therefore correlated with the individual behaviour of adults (Koenig and Ousterhout, 2018). In addition, the propensity of dispersal, i.e., dispersal range and activity, has been shown to be related to behavioural syndromes; thereby contributing to population dynamics and very likely to differential gene flow between populations (Cayuela et al., 2016, 2020; Denoël et al., 2018).

The general environmental factors and morphological constraints and therefore the timing of emigration might be similar between sexes, but how is it about the propensity of dispersal? In many amphibians, sex-dependency has been shown, however, there does not seem to be an overall trend towards male-biased or female-biased dispersal and in some species a sex difference is missing completely (reviewed in Cayuela et al., 2020). The hypothesis would be that in species with high resource partitioning by males (e.g., territorial behaviour), dispersal is female-biased and male-biased, if the mating system is based on female choice. However, this simple prediction does not seem to be supported by field data (Smith and Green, 2006). In general, dispersal capacity may be underestimated as such studies are always limited by the studied areas (Smith and Green, 2005). Dispersal differences have been shown in directly developing salamanders, where gene flow is based mainly of the movement of males (Liebgold, Brodie III, and Cabe, 2011; Helfer, Broquet, and Fumagalli, 2012). Female-biased dispersal has been suggested for the pool breeding bull frog and the European common frog (Austin et al., 2003; Palo et al., 2004).

As I have argued above, the emigration direction is influenced by the topography surrounding the breeding habitat. However, does an inherited component exist? This topic received limited attention in the amphibian literature, nevertheless it is worth to discuss. Evidence from other taxa, e.g., migrating birds, suggests the possibility of an inherited dispersal direction (see also the marine sand hopper example above). Juvenile migratory birds, such as blackcaps, manage to find their winter habitats without learning a direction and exhibit the seasonal appropriate responses (e.g., towards southern directions) even when they are reared in the laboratory. It has been shown that crossing of blackcaps from two populations with different winter habitats and therefore different dispersal directions, leads to offspring with an intermediate orientation response (Helbig, 1991; Helbig et al., 1994). These early studies were followed up by more detailed genetic analyses (for a review on this topic see Merlin and Liedvogel, 2019). However, the genetic underpinning of juvenile dispersal is still poorly understood. Amphibians would provide a suitable species to study such phenomena.

To the best of my knowledge, only Miaud et al. (2005) tested the hypothesis that the emigration direction is genetically inherited in amphibians. First, they analysed dispersal movements of European common frog froglets from different populations. Second, they identified two populations – at the northern and southern edges of a 4 km long lake – with highly directed dispersing directions when tested in arenas (towards North and South, respectively). They then experimentally crossed males and females from these populations and tested the dispersal orientation of the offspring. The obtained responses differed significantly from the parental orientation and approximated an intermediate orientation pattern (towards West). It would be highly valuable to continue this line of research and test, if dispersal direction can be, at least partially, inherited. This question could be explored using a mix of behavioural experiments and genetic techniques. For example, in an experimental set-up (similar to Miaud et al., 2005) one could identify amphibians with specific emigration direction and selectively breed these over several generations. If the emigration direction would have a genetic component, the artificial selection should lead to a clear selection pressure on specific genomic regions, in addition one would expect that the offspring will choose increasingly consistent emigration direction. An alternative approach would be to identify several populations of the same species with unidirectional and known emigration directions and test for correlations of allele frequencies and/or gene expression with direction (see discussion of potential approaches in Merlin and Liedvogel, 2019). If genomic regions of interest were identified and laboratory colonies are available, the next step could be to genetically manipulate the target genes and thereby alter the emigration directions (the gene editing tool RISPR/Cas9 is already available for amphibians, e.g., Wang et al., 2015).

**Figure 1:**
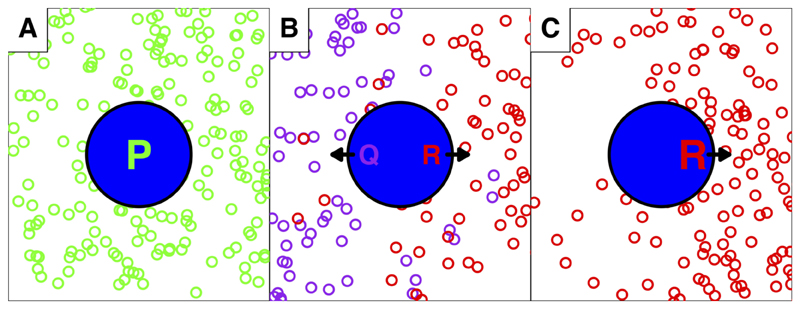
Three hypothetical examples of amphibian populations are shown, a ‘pond’ (breeding habitat) is shown in the centre, symbolized by a blue circle; open circles represent individual amphibians, the colours correspond with the population letter (“P” = green, “Q” = purple and “R” = red). Two assumptions for this scenario are the following: demographic estimates are calculated from a capture-recapture effort using adults at the breeding site, and that the (former) emigration direction of adults influences their offspring’s emigration direction. Individuals of population “P” in the first example (A) are emigrating randomly from their natal pond, and thereby distributing equally spaced. However, in the second example (B) two subpopulations (“Q” and “R”) exist with two emigration directions towards opposite sides. Overall, again the distribution of individuals appears equally spaced, however, in reality it is composed of two underlying subpopulations. Further inspection of this example would reveal that the individual density towards the North and South is slightly sparser then towards East and West, anyway, the differences are minor. In the third example (C) distribution “Q” went extinct and only “R” remained. Despite the same number of animals emigrating, the spatial distribution is very unequal between the East and West side of the pond. If we assume for all three examples that population density estimates were based on measures from the pond in the centre, estimates will agree well with the ‘true’ situation for the populations in A and B. In contrast, such measures would fail to predict animal presence for C. Distributions were calculated using R (R Core Team, 2020). For individuals of population “P” a random sample along x and y-axes was plotted, for population “Q” and “R” the individual positions followed a normal distribution on the x-axis, with the means left (“West”) and right (“East”) of the ponds as well as the centre of the y-axis for both. For plotting I used the packages grid (R Core Team, 2020), shape (Soetaert, 2020), ggplot2 (Wickham, 2016), ggplotify (Yu, 2020) and cowplot (Wilke, 2020).

Such directionality of emigration (learned or genetically inherited) could have important consequences for the spatial distribution of a population. For instance, if there were directional tendencies of amphibians emigrating from their breeding habitat, which may translate in a re-occurring migratory route, this would change their distribution in the landscape. Essentially, part of the surrounding area of a breeding habitat might be ‘avoided’ by the animals, due to the initial – potentially inherited – emigration direction, independent of and adding onto other known factors (e.g., distance, landcover, microclimate etc.). Therefore, demographic estimates (usually obtained by (re-)capturing adults at the breeding sites), such as population size, would in reality be unequally distributed around the breeding habitat while modelling approaches would assume equal distribution (fig. 1). This could lead to over- as well as underestimation of the population at different areas of the landscapes. Importantly, the directionality could be an individual’s trait, which may also shape following generations (either through inheritance or habitat choice) and may not be immediately related to spatial parameters such as land-cover. In recent methodological developments of population statistics, spatial parameters can be incorporated in population modelling (see for example Efford and Fewster, 2013; Efford and Schofield, 2020). Such spatially explicit analyses have already shown to provide new insights and overcome some of the shortcomings of more traditional approaches (e.g., detect difference between mortality and emigration). Adding a directional tendency of individuals or certain subpopulations, could further improve the prediction accuracy of spatially explicit population models.

## Influence of natal habitat cues on philopatry

It is well established that many adult amphibians display site fidelity, they revisit the same breeding habitats every year (Reading, Loman, and Madsen, 1991; Sinsch, 1992; Kupfer and Kneitz, 2000). In some species, in the neotropics, males occupy breeding territories to which they also home after experimental displacement (Ringler, Ursprung, and Hödl, 2009; Pašukonis et al., 2013). In addition, to a varying degree amphibians show philopatry, they prefer their natal habitat over others (Semlitsch, 2008). This means that a large percentage of animals from one generation will return to their natal habitat for breeding; for example, in a study using marbled salamanders 91% first-time breeders returned to their natal ponds (Gamble, McGarigal, and Compton, 2007). The strength of philopatry likely depends on many factors including habitat stability, as it has been shown in several *Bombina variegata* population analyses (Cayuela et al., 2016, 2020; Boualit et al., 2019). For example, anthropogenic disturbance increases exploratory and dispersal behaviour, an effect that appears to be genetically determined (Cayuela et al., 2020). However, it is still understudied which cues animals can use to distinguish between natal and non-natal habitats. It has been speculated what such behaviour could be based on, for example, animals could prefer habitats with stimuli similar to the natal ones (Davis and Stamps, 2004). However, Ousterhout et al. (2014) tested this hypothesis and found no preference for a natal substrate type in mesocosm experiments. Philopatry could also be supported by imprinting on the chemical cues present during their larval stages. In fact, several anurans are able to learn to differentiate between their native pond water and a control (Ogurtsov, 2003; Shakhparonov and Ogurtsov, 2003). How and if this contributes to philopatry remains untested, although the importance of this questions for e.g., relocation efforts of protected species. I hypothesize that such imprinting is based on a variety of factors, similarly to the many orientation cues that can be used by amphibians. Testing only one factor alone might be insufficient. Such factors could include chemical cues, landscape features, water body topography and geographic location (e.g., magnetic imprinting). To test this issue, one would have to combine laboratory and field experiments, preferably using animals from well monitored populations, where the origin of individuals is, at least partially, known. Then one could test a preference for natal habitat types/landscape features in the field, as well as test preference for natal chemical cues, magnetic values, and water body features in the laboratory.

## Proposed research questions and concluding remarks

There exist a disconnect between our understanding of the larvae and juvenile amphibian spatial behaviour in their natal habitat and its influence on emigration. Furthermore, the processes shaping the emigration direction are poorly understood, despite its importance. I propose five research hypotheses which could provide valuable insights in amphibian population and migration biology:

1. Movement along the y-axis is (mainly) a predator avoidance behaviour, perturbation of such orientation system will increase larval predation.
2. There is an inherited component of juvenile emigration directions, which functions in an interplay with learned behavioural responses and passive factors such as physical barriers, to shape the spatial distribution of adult (pond breeding) amphibians.
3. Such (inherited and learned) directional factors can be incorporated in population models and will increase the explanatory value of such models in terms of predicting spatial distribution.
4. Amphibians optimize the timing of their individual emigration based on a fitness-trade-off between internal factors (e.g., body condition/growth rate) and dispersal risk.
5. Cues of the natal habitat influence the general migration behaviour of amphibians, i.e., high quality habitats correlate with lower dispersal propensity and higher site fidelity.

I hope that this short paper can spark some interest of fellow researchers to follow up some of these rather specific but important issues in amphibian ecology. I am confident that this will not only improve our understanding, but also help conservation efforts around the world.
